# Navigating Adult Disability Services by Latino Families of Youth with Autism

**DOI:** 10.1007/s10882-025-10019-7

**Published:** 2025-04-25

**Authors:** Gabriela Herrera Espinosa, Meghan M. Burke, Robert M. Hodapp

**Affiliations:** Department of Special Education, Peabody College, Vanderbilt University, Nashville, TN USA

**Keywords:** Autism, Adult service-access, Latino Caregivers, Knowledge, Advocacy Training

## Abstract

Compared to the general population, Latino families of youth with autism face unique barriers in accessing adult disability services. Barriers include limited access to resources in Spanish and discrimination. By characterizing specific barriers to advocacy abilities, including knowledge about adult disability services, advocacy skills and comfort, and empowerment, targeted interventions can be developed to improve service access among Latino families. In this study, we examined the responses of 45 Latino caregivers (primarily mothers) before completing a 12-week advocacy program. Results demonstrated significant positive correlations between comfort in advocating for services and their sense of empowerment. Greater proficiency with the English language positively correlated with advocacy skills and comfort. Compared to empowerment within systems (e.g., service delivery systems, the community/political system), participants demonstrated significant strengths in family empowerment. Findings from this study shed light on the importance of designing culturally responsive resources for Latino families.

For youth with disabilities, transitioning to adulthood can be challenging. As they exit high school, youth must adjust to experiences beyond the academic setting, from finding an appropriate job to pursuing higher education opportunities to living independently (Taylor et al., 2017). Many of these accomplishments are aided by transition planning, a set of procedures mandated by the Individuals with Disabilities Education Act (IDEA). Such transition planning, in turn, involves the participation of both the students and their families, with IDEA specifically stating that transition planning requires family involvement. Indeed, participation of family members may be especially important during transition planning, as families constitute the primary advocates for services for their offspring with disabilities (Burke et al., 2019).

Although family involvement is central in transition planning, many families remain unfamiliar with services and supports for their offspring (Aleman-Tovar et al., 2022). In particular, families of youth with autism report struggling to access services after high school, as their offspring no longer receive services under IDEA (Taylor et al., 2017). Even compared to their peers with other types of disabilities, young adults with autism are less likely to receive services after graduation, including such services as speech, physical, and occupational therapy (Laxman et al., 2019).

To foster family participation in transition planning and access to adult services, several programs have targeted improving parent knowledge of adult services, empowerment, and advocacy skills. For example, after attending the Volunteer Advocacy Program-Transition (VAP-T), a 12-week advocacy program for families of transition-aged youth with autism, intervention participants (compared to those in the waitlist-control group) demonstrated improved knowledge of adult services, advocacy skills, and empowerment (Taylor et al., 2017). Further, intervention participants demonstrated greater access to services for their offspring. It seems that knowledge, empowerment, and advocacy are key predictors of service access for families of transition-aged autistic youth.

But such connections may not be the same for all families. Consider Latino families. Latino refers to a person of Mexican, Cuban, Puerto Rican, Central or South American, or other Spanish origin or culture, regardless of race (U.S. Census Bureau, 2022). Compared to their non-Latino, white peers with autism, Latino youth with autism are less likely to receive adult services (Schott et al., 2021). Such lower levels of service access may relate to systemic barriers. Describing the experiences of Latino families of transition-aged youth with autism, for example, Aleman-Tovar et al. (2023) found that Latino families reported discrimination, limited information in Spanish, and the absence of culturally responsive materials. Each barrier may influence the degree to which Latino parents are able to know about, advocate for, and feel empowered to attain necessary services for their offspring.

As a first step in understanding systemic barriers, it would help to identify the specific types of knowledge, empowerment, advocacy, and service access that are especially difficult for Latino families. By identifying the types of knowledge, empowerment, advocacy and service access, areas of weakness can be targeted for intervention and areas of strength can be leveraged. Regarding knowledge, we need to discern whether Latino families show different levels of knowledge of school-based services versus services provided by community agencies (Young et al., 2016), or whether Latino families differentially know about some (vs. other) types of adult services (e.g., Medicaid waivers, health insurance, Social Security Disability Insurance, vocational rehabilitative services; Taylor et al., 2017). It may also be the case that Latino parents experience different levels of empowerment with respect to their family (i.e., feeling empowered to know the needs of one’s child), as compared to navigating the service delivery system or making systemic changes in the community/political system (Koren et al., 1992). Similarly, are Latino parents more comfortable performing certain advocacy activities and do they find some services easier or harder to access? By identifying the types of knowledge, empowerment, advocacy, and services that are lower among Latino families, interventions can be developed to improve areas of specific need.

An additional issue concerns how knowledge, empowerment, and advocacy relate to one another—are parents who are more knowledgeable about adult services more likely to rate themselves higher on empowerment or advocacy comfort? And, ultimately, we need to know the degree to which one’s knowledge, advocacy comfort, and empowerment relate to service access. Some studies suggest that empowerment (versus knowledge and advocacy) most strongly relates to service access (Taylor et al., 2017). Other studies suggest that empowerment (versus knowledge) most strongly relates to advocacy activities (Li et al., in press). To date, no studies have examined these connections for Latino families. It is important to characterize the relations among these constructs to identify the key mechanisms of action that should be targeted in interventions to improve service access.

Finally, it is important to consider the connections to a family’s linguistic status. As most school-based meetings, documents, and services are provided in English, Latino youth and their families report barriers in interacting with service providers due to language differences (Francis et al., 2018). Granted, language is but one aspect of acculturation, which entails “all the changes that arise following contact between individuals and groups of different cultural backgrounds” (Sam, 2006, p. 11). Still, at a basic level, we can begin to understand the relation between acculturation and service access by examining the relations between language and service access and related constructs. This information can be used to target interventions to individuals who are prone to lower levels of service access.

This study, then, extends prior work on knowledge, advocacy, and empowerment to Latino families. To this end, we examined two research questions: (1) Which aspects of knowledge about services, comfort-skills with advocacy activities, empowerment, and service access are especially difficult for Latino families? And (2) To what extent do knowledge, advocacy, empowerment, English-language skills, and service access relate to one another?. Drawing from the literature about Latino families and the role of values such as *familismo*, which prioritizes the family’s needs over the individual (Steidel & Contreras, 2003), we hypothesized that Latino families would report greater comfort in participating in family-oriented activities compared to involvement in service and political activities (Aleman-Tovar et al., 2022). Additionally, we hypothesized that Latino families’ knowledge and comfort in advocating for their offspring would relate to families’ language proficiency in English (Xu et al., 2022).

## Method

### Participants

The 45 participants of this study were mostly mothers (*n* = 38, 84%), of Mexican origin (*n* = 38, 86%), and married (*n* = 31, 69%). Slightly less than half (*n* = 20, 47%) reported annual incomes of less than $29,000. Approximately 53% (*n* = 24) of participants completed high school or some college. The offspring of the participants were mostly male (*n* = 37, 82%) and ranged in age from 12 to 28 (*M* = 14.97, *SD* = 5.04); all were reported by caregivers to have autism. See Table 1.

**Table 1:** Participant demographics

Characteristics	% (*n*)
Gender: Female	90.7% (39)
Role	
Mother Father Legal Guardian Other	84.4% (38) 8.9% (4) 2.2 (1) 4.4% (2)
Marital Status	
Married Never married Separated Divorced Widowed	68.9% (31) 8.9% (4) 13.3% (6) 4.4% (2) 4.4% (2)
Race/ethnicity	
Mexican Puerto Rican Central American South American Other	86.4% (38) 4.5% (2) 2.3% (1) 4.5% (2) 2.3% (1)
Educational Background	
Some high school High school or some college College/Graduate School degree	22.2% (10) 53.4% (24) 24.4% (11)
Annual Income	
Less than $15,000 Between $15,000–29,000 Between $30,000–49,000 Between $50,000–69,000 Between $70,000–99,000	23.3% (10) 23.3% (10) 37.2% (16) 4.7% (2) 11.6% (5)

To participate in this study, potential participants needed to meet several inclusion criteria. Specifically, participants needed to: be at least 18 years of age, reside in Illinois, and be raising in their home an autistic child who was at least 12 years old (we chose 12 as recent studies emphasize the importance of starting parent, offspring, and school discussions of transition in the early teen years). All participants also agreed to enroll in a 12-week advocacy program for Spanish-speaking families.

### Recruitment

To help recruit participants for this study, we relied on *personalismo*, which entails building rapport and a relationship based in trust between potential participants and researchers (Magaña, 2020). Specifically, participants were recruited through established connections between the authors and Parent Training and Information Centers, disability agencies in Illinois (e.g., The Arc of Illinois), and word of mouth in parent support groups. A recruitment flyer with information about the study was disseminated in English and Spanish. Each participant received a $20 gift card after completing the survey.

### Procedures

There were no potential conflicts of interest in this study. All procedures performed in studies involving human participants were in accordance with the ethical standards of the institutional and/or national research committee and with the 1964 Helsinki declaration and its later amendments or comparable ethical standards. The study received approval from the University Institutional Review Board to conduct research with human subjects. Each participant provided informed consent before participating in the study.

To ensure that all participants met the inclusionary criteria for the study, interested individuals completed a screening form. If the individual met the inclusionary criteria, the prospective participant then provided informed consent and completed the survey (which reflects the measures listed below). While a hard copy option was available, all participants completed the survey via REDCap, a web-based survey platform. The survey was available in English and Spanish. The survey was open for three months. Following the completion of all measures (see below), data were downloaded from REDCap into SPSS.

### Measures

**Knowledge of Adult Services**. The *Adult Services Knowledge Scale* (Taylor et al., 2017) consisted of 22 multiple-choice questions related to knowledge about adult services (e.g., Vocational Rehabilitation, Social Security, Medicaid, Medicare). Example items included: “How is a youth eligible for vocational rehabilitation services?” and “How can an individual be eligible for a housing voucher?” Each item had four potential response options, one of which was correct. Each response was recoded as incorrect (0) or correct (1). For this study, the Kuder-Richardson coefficient was 0.81.

**Advocacy comfort**. The *Advocacy Skills and Comfort Scale* (Taylor et al., 2017) is a 10-item scale that measures how comfortable and skilled participants perceive themselves to be in advocating for their youth. An item was: “How able are you to advocate for your child’s needs in trying to get adult services?”. Each item was rated on a 5-point Likert scale from *not at all* to *excellent*. Scores were summed and averaged across the 10 items. The Cronbach’s alpha was 0.91.

**Advocacy activities**. The *Advocacy Activities Scale* (Li et al., 2024) is a 16-item measure to gauge the extent to which parents engaged in advocacy behaviors. Items included the degree to which the parent has “Searched the internet to find agencies and/or services to meet your child’s needs” and “Tried to get media attention about disability services.” Rated on a 5-point Likert scale (from *not at all* to *very often)*, items were summed and averaged for this study. The Cronbach’s alpha was 0.94.

**Empowerment**. The *Family Empowerment Scale* (Koren et al., 1992) measures empowerment for parents of individuals with disabilities. Each item was rated on a 5-point Likert scale from 1 (*strongly disagree*) to 5 (*strongly agree*). Comprised of 34 questions, participants rate the degree to which they felt empowered in three subscales: family, services, and community/political systems. The Family subscale has 12 items (e.g., “I feel confident in my ability to help my son/daughter grow and develop.”). The Cronbach’s alpha was 0.88. The Service system subscale has 12 items related to attitudes, behaviors, and knowledge about services (e.g., “I know what steps to take when I am concerned my son/daughter is receiving poor services.”). The Cronbach’s alpha was 0.88. The Community/Political subscale included 10 items related to collaborating with policymakers, agencies, and community members (e.g., “I help other families get the services they need”). The Cronbach’s alpha was 0.85.

**Services Inventory**. The *Services Inventory* (Burke & Fulton, 2023) was comprised of 10 adult disability services. For example, an item was: “Is your son/daughter *currently* receiving SSI (supplemental security income)?” If the participant responded they were not receiving the service, they were asked if they *needed* the service (i.e., unmet service need). For currently receiving services and unmet service needs, responses were dichotomous: “no” (0) or “yes” (1). If the youth was not receiving the service but currently needed it, participants were asked “Did you not know about the service and/or how to get this service?”. Responses were “no” or “yes”. Items were summed to obtain the total number of received and needed services. Services included: health insurance, Supplemental Nutrition Assistance Program (SNAP), SSI, Social Security Disability Insurance (SSDI), Medicaid Waivers, legal protection, Medicaid Long Term Services and Supports, Vocational Rehabilitation, Special Needs Trust/ABLE Accounts, and the housing choice vouchers. The Kuder-Richardson coefficient was 0.78.

**Acculturation Scale**. The *Language Acculturation Scale* (Felix-Ortiz et al., 1994) consisted of three items about the respondent’s comfort in reading, speaking, and writing in English. Participants rated each option on a 4-point Likert scale, ranging from 1 (*Poorly*) to 4 (*Excellent*). A composite measure was created by summing the individual items with higher scores indicating greater proficiency in English.

### Data Analysis

There were no missing data; all data were normally distributed. To identify which aspects of knowledge about services, comfort with advocacy, empowerment, and service access were especially difficult for Latino families, we performed repeated measures ANOVAs for variables with non-dichotomous response options (i.e., Advocacy Skills and Comfort, Advocacy Activities Scale, and three types of Empowerment) and McNemar’s testes for variables with dichotomous response options (i.e., Knowledge of Adult Services and Services Inventory). Effect sizes were conducted via Cohen’s *d*. For all analyses, our goal was to identify items that were more (versus less) known or more likely to be present. To examine the relations among knowledge, advocacy, empowerment, service access, and English-language proficiency, we performed Pearson correlations.

## Results

### Difficulty Level of Knowledge, Advocacy, Empowerment, and Service Access

**Knowledge**. Overall, participants demonstrated low levels of knowledge of adult disability services, with some individual items easier and some harder for participants. Out of 22 knowledge items, the average number of correct items was 5.45, or 24.75% (range from 4.4 to 53.3%). Comparing across items, correct responses were more often noted to person-centered planning (53.3%) and guardianship (51.1%); items with fewer correct responses included those related to eligibility criteria for SNAP (4.4%), Medicaid (8.9%), and post-secondary education (8.9%). We identified whether participants were “high responders” (i.e., answered more then 25% of the questions correctly) or “low responders” (i.e., less than 25% correct). See Table 2.

**Table 2:** Knowledge high and low scores

Item	Correct Response % (*n*)	High or Low
**Person-centered planning**: Which of the following requires person-centered planning (i.e., wherein all decisions are based on the strengths, needs, and preferences of the person with a disability)?	53.3% (24)	**H**
**Decision-making**: Which of the following is the MOST restrictive form of decision-making support that could limit the choices of the person with a disability?	51.1% (23)	**H**
**Employment**: When an employer and worker design a job, it is called…	44.4% (20)	**H**
**Special Needs Trusts**: Who has the sole authority to make decisions about the use of a special needs trust?	40% (18)	**H**
**Housing**: How can an individual be eligible for a housing voucher?	35.6% (16)	**H**
**Employment**: What paperwork is necessary for transition-aged youth to qualify to receive Workforce Innovation Opportunity Act services?	35.6% (16)	**H**
**Employment**: Which of the following options describes employment in an inclusive work setting with ongoing support services for individuals with disabilities?	35.6% (16)	**H**
**Housing**: What is public housing?	28.9% (13)	**H**
**Vocational Rehabilitation**: How is a youth eligible for vocational rehabilitative services?	26.7% (12)	**H**
**Medicaid**: When moving to a new state, what do you need to do to continue to access services with the Home and Community-Based Services Waivers?	22.2% (10)	**L**
**Postsecondary education**: Select two pieces of policies that may help pay for students with disabilities to receive post-secondary education.	22.2% (10)	**L**
**Special Needs Trusts**: An ABLE account can hold…	22.2% (10)	**L**
**Health**: The Affordable Care Act (also known as Obamacare) allows children to stay on their parents’ insurance until what age?	20% (9)	**L**
**Medicaid Waiver**: Which of the following CANNOT be paid for by a Home and Community-Based Services Medicaid Waiver?	20% (9)	**L**
**Health**: Which of the following benefits automatically qualifies your child for Medicare health insurance?	15.6% (7)	**L**
**Supplemental Security Income (SSI)**: Which of the following is NOT a requirement for Supplemental Security Income (SSI)?	15.6% (7)	**L**
**Social Security Disability Insurance (SSDI)**: How can an individual qualify for Social Security Disability Insurance (SSDI)?	13.3% (6)	**L**
**SSI**: What is substantial gainful activity?	20% (9)	**L**
**SSI**: With respect to Supplemental Security Income (SSI), what are your options if your young adult cannot handle their finances?	20% (9)	**L**
**Post-secondary education**: Select the two pieces of legislation that enable students with disabilities to receive accommodations in post-secondary education.	8.9% (4)	**L**
**Medicaid**: With respect to long-term services and supports, which of the following is an alternative to Home and Community-based Services Waivers?	8.9% (4)	**L**
**Supplemental Nutrition Assistance Program (SNAP)**: How does an individual qualify for the Supplemental Nutrition Assistance Program (SNAP)?	4.4% (2)	**L**

**Advocacy Activities and Skills-Comfort**. For both advocacy measures, significantly easier and harder items emerged. Put simply, some advocacy activities were more common than others, *F* (15, 660) = 26.63, *p* < 0.001, with participants reporting feeling like they “*Searched the internet to find agencies and/or services to meet your child’s needs*.” (*M* = 3.26, *SD* = 1.04) more than they “*Tried to get media attention (e.g*., *newspaper*, *television*, *radio) about disability services*” (*M* = 1.33, *SD* = 0.83). Notably, a “1” refers to “not at all” conducting the advocacy activity whereas a “3” indicates that the advocacy activity is conducted often. Within the Advocacy Skills and Comfort Scale, items again differed, *F* (9, 396) = 8.68, *p* < 0.001, with higher-rated items concerned caregiver involvement in meetings with professionals (e.g., “*How comfortable are you in your ability to effectively participate in meetings with providers/agencies/professionals*”) (*M* = 3.16, *SD* = 1.21) as compared with their knowledge and rights in navigating adult services (e.g., “*How knowledgeable do you think you are about your rights in the adult service system?* (*M* = 2.11, *SD* = 0.91). See Table 3.

**Table 3:** Empowerment and advocacy high and low scores

Scale	Average score	Item	Mean (SD)	High or Low
Family Empowerment	**3.85**	*I feel I am a good parent*	4.43 (0.779)	**H**
		*I feel my family life is under control*	3.19 (1.26)	**L**
Service System Empowerment	**3.62**	*When necessary*, *I take the initiative in looking for services for my son/daughter and family.*	4.09 (0.985)	**H**
		*My opinion is just as important as professionals’ opinions in deciding what services my son/daughter needs.*	2.76 (1.43)	**L**
Community/Political Empowerment	**2.40**	*I believe that other parents and I can influence on services for people with disabilities.*	4.11 (0.875)	**H**
		*I get in touch with my lawmaker when important bills or issues concerning people with disabilities are pending*	1.91 (1.21)	**L**
Advocacy Activities	**2.24**	*Searched the internet to find agencies or services to meet your child’s needs.*	3.27 (1.03)	**H**
		*Tried to get media attention (e.g.*, *radio) about disability services.*	1.37 (0.853)	**L**
Advocacy Skills and Comfort	**2.82**	*How able are you to effectively participate in meetings with providers/professionals?*	3.13 (1.204)	**H**
		*How knowledgeable do you think you are about your rights in the adult service system?*	2.13 (0.909)	**L**

**Empowerment**. Similar differences were noted for empowerment, with differences across subscales and items within each subscale. Across subscales, participants reported significantly higher scores on Family Empowerment (*M* = 46.46, *SD* = 7.39) compared to Service System Empowerment (*M* = 43.43, *SD* = 8.85) and Community Empowerment (*M* = 34.06, *SD* = 7.39), *F* (2, 68) = 180.03, *p* < 0.001. Exceptionally large effect sizes emerged across these three empowerment domains, with Community Empowerment much lower than both Services Empowerment (*ES* = 1.17) and Family Empowerment (*ES* = 1.75); Family Empowerment showed a medium effect size compared to Services Empowerment (*ES* = 0.45). Notably, higher scores indicate that the participant had more agreement with the statements of empowerment.

Items within each of the three Empowerment domains also showed significant highs and lows. Within the Family Empowerment Subscale, the item “*I feel I am a good parent*” was rated higher than “*I feel my family life is under control*”, *t* (44) = 5.90, *p* < 0.001. In the Service System Subscale, the item “*When necessary*, *I take the initiative in looking for services for my son/daughter and family*” was rated higher than “*My opinion is just as important as professionals’ opinions in deciding what services my son/daughter need*s” *t* (44) = 5.82, *p* < 0.001. In the Community/Political Subscale, participants reported a higher sense of empowerment for items related to their impact on service access (e.g., “*I believe that other parents and I can influence services for people with disabilities*), compared to reaching out to lawmakers in their community (e.g., “*I get in touch with my lawmaker when important bills or issues concerning people with disabilities are pending” or “I have a good understanding of the services system that my son/daughter is involved in*; both p’s < 0.001).

**Service Access**. In regard to services, a higher percentage of individuals received health insurance (84%; *n* = 31), and SNAP (46%; *n* = 17) compared to Vocational Rehabilitation (8%; *n* = 3), Special Needs Trust and/or ABLE accounts (3%; *n* = 1), and the Housing Voucher (3%; *n* = 1). Moreover, 51.4% (*n* = 19) of participants indicated that not knowing about a service or how to receive a service was a barrier to accessing adult disability services. See Table 4.

**Table 4:** Barriers to accessing services

Type of Service	Receives the service % (*n*)	Needs the service % (*n*)	Does not know whether they need the service % (*n*)	Limited knowledge impedes service access
Health insurance	83.8% (31)	16.7% (1)	0	No
SNAP	45.9% (17)	45% (9)	8.1% (3)	Yes
SSI	37.8% (14)	47.8% (11)	8.1% (3)	Yes
Medicaid Waiver	27% (10)	57.7% (15)	18.9% (7)	Yes
Legal protections	24.3% (9)	18.5% (5)	5.4% (2)	Yes
SSDI	13.9% (5)	57.7% (15)	53.3% (8)	Yes
Long-term Services and Supports	13.5% (5)	41.9% (13)	18.9% (7)	Yes
Vocational Rehabilitation	8.3% (3)	40% (12)	18.9% (7)	Yes
Special Needs Trust	2.7% (1)	56.3% (18)	35.1% (13)	Yes
Housing Voucher	2.7% (1)	12.1% (4)	8.1% (3)	Yes

### Correlates of Knowledge, Advocacy, Empowerment, Language Proficiency, and Service Access

Significant, positive correlations emerged between the scales of Advocacy Skills-Comfort and multiple measures, notably Advocacy Activities and several areas of Empowerment (especially Community/Political Empowerment). Several of these correlations were in the moderate to high range (*r’*s from 0.43 to 0.64). The only significant correlation with Total Services Received was Advocacy Activities (*r* = 0.39); relations with Knowledge, Advocacy Skills-Comfort, and the three types of Empowerment were all non-significant. Greater English proficiency was highly correlated with advocacy skills and comfort, *r* (43) = 0.67, *p* < 0.001. See Table 5.

**Table 5:** Correlations between scores across constructs

	1	2	3	4	5	6	7	8
1. Knowledge	-							
2. Advocacy activities	0.38*	-						
3. Advocacy skills and comfort	0.43**	0.63**	-					
4. Family empowerment	0.14	0.33*	0.47*	-				
5. Service system empowerment	0.20	0.39**	0.52**	0.89**	-			
6. Community/Political empowerment	0.25	0.46*	0.64**	0.89**	0.86**	-		
7. Total services received	0.17	0.39*	0.18	0.17	0.23	0.29	-	
8. Overall English Proficiency	0.31*	0.34*	0.67**	0.25	0.28	0.33*	0.18	-

*p < 0.05, **p < 0.01

## Discussion

Although researchers are beginning to identify the supports needed to help transition-aged youth with autism, few studies have explored service access for Latino families. As one of the first studies to examine the knowledge, advocacy, empowerment, language proficiency, and service access among Latino families of transition-aged youth with autism, this study had three main findings, each with research and practical implications.

Our first finding related to specific aspects of knowledge, advocacy, empowerment and service access for Latino families. While Latino caregivers reported themselves most empowered in terms of their own child with disabilities, by far the lowest ratings related to community/political systems, with scores below both family and service-system empowerment. Strong feelings of family empowerment (versus the service delivery system and community/political system) align with extant literature. A key value of Latino culture is familism, wherein the priority is the family and not the individual (Steidel & Contreras, 2003). Thus, it is unsurprising that a tremendous strength among Latino families is family empowerment. This finding underscores the familial capital within Latino families (Yosso, 2005) and provides a specific way to apply a strengths-based (as opposed to deficit-focused) framework to intervention research with Latino families.

Perhaps conversely, the finding also aligns with studies suggesting that Latino families struggle with systems-related empowerment, including those systems related to service delivery, the community, and political institutions. Prior research has identified several systemic barriers facing Latino families including racism, discrimination, and cultural unresponsiveness (Aleman-Tovar et al., 2022; Francis et al., 2018). Perhaps as a result of structural barriers, Latino families feel less empowered when navigating systems. In considering this finding, there may need to be policy changes to improve empowerment among Latino families. Specifically, instead of trying to intervene with Latino families to improve their systems empowerment it may be more appropriate to change the system which is seemingly reinforcing inequities. Such changes could include making information available in English and Spanish for Latino families.

A second finding concerned correlations among constructs. The most striking relations occurred between Advocacy Skills and Comfort and the total scores of advocacy activities, the three types of empowerment, and knowledge. Such connections align with prior research with primarily white families of transition-aged youth. For example, in a study of 186 parents of transition-aged youth with autism (most of whom were non-Latino, white families), Li et al. (in press) found that advocacy activities were closely tied to empowerment. Our study suggests that the relation between advocacy and empowerment is also present among Latino families.

Conversely, few advocacy constructs related to actually attaining disability services, with the sole connection to services received involving greater amounts of advocacy activities (and even this correlation was relatively low; *r* = 0.39). Future research would seem needed to delve more closely into the connections—and directionality—among advocacy activities, empowerment, and service access. Longitudinal research could help determine the ways in which Latino families develop advocacy skills (e.g., knowledge, empowerment, comfort with advocacy) and whether such skills lead to improved access to services.

Our last finding related English proficiency to advocacy skills-comfort. Across the sample, there was a strong relation between English proficiency and advocacy skills-comfort. On its surface, this finding is not surprising. Latino families report struggling to advocate given limited Spanish-language resources (Wilt & Morningstar, 2018), with those families least proficient in English facing greater challenges in advocacy (Zuckerman et al., 2017). Altogether, there is a need to create more resources in Spanish to help Latino families access services, which will also help foster comfort with advocacy skills.

### Implications for Research and Practice

Overall, these findings have several implications. In terms of research, we need to examine how the barriers faced by Latino families relate to systemic barriers. In this study, many participants judged their own opinions as less important than the views of professionals; they also provided exceptionally low ratings to the advocacy activity of contacting lawmakers. It may be that, in navigating U.S. service-delivery systems, Latino participants experience many differences from receiving services within their home country/culture. As Whitehead et al. (2020) noted, navigating two cultures can bring strengths (e.g., higher self-esteem, improved family relationships) and stressors (e.g., difficulty switching between two sets of cultural norms). By understanding the impact of navigating two service delivery systems, we can develop a more holistic understanding of the experiences of Latino families of transition-aged youth with autism.

More generally, participants also reported a lack of knowledge about the U.S disability service system. Granted, the American disability service-system is notoriously hard to understand, with many describing such services as complicated, unconnected, and ever changing (Timmons et al., 2004). In response to this state of affairs, specific services have arisen, especially disability-related information-and-referral (Hodapp et al., 2018) and navigator programs (Broder-Fingert et al., 2020). Given the sample’s low scores on disability-service knowledge and the general lack of Spanish-language material, additional, targeted information would seem necessary to provide better roadmaps to service for Latino parents. On a positive note, successful, evidence-based programs in Spanish have been initiated for Latino families to access services (e.g., Parents Taking Action, Magaña et al., 2020). Such programs now need to be replicated and disseminated nationally.

When developing advocacy programs, researchers and practitioners should also target improving community and political empowerment and systemic advocacy. As often noted, Latino families face systemic barriers in accessing services, most notably discrimination and racism in looking for services (Xu et al., 2022). In response to such barriers, culturally-responsive services are necessary. But such cultural responsivity likely extends beyond providing the program in Spanish. As Wilt and Morningstar (2018) note, interventions that consider the unique linguistic *and* cultural needs of racial minority families lead to stronger collaborations among service providers, professionals, and families. Such programs also likely necessitate designing interventions in collaboration with the Latino families who will be the program’s recipients (Kuhn et al., 2020).

### Limitations

Although this study contributes to the understanding of Latino families of transition-aged youth with autism, it is important to note several limitations. First, all participants lived in the same Midwestern state. Future research should consider the experiences of Latino families across other regions in the United States, including rural, suburban, and urban settings (Francis et al., 2020). Second, because the majority of participants were of Mexican origin or descent, the findings may not generalize to Latino families of other heritages. Third, this study was cross-sectional; thus, the direction of effects cannot be determined. Finally, all participants expressed interest in participating in an advocacy program; thus, the participants may not be generalizable to the general population of Latino families.

Still, even considering these limitations, these findings shed light on the experiences and needs of Latino families of youth with autism. By examining the impact of language and knowledge barriers—as well as their correlates with empowerment, service access, and advocacy—we begin to understand the supports Latino families need to become active participants in their offspring’s transition to adulthood.
